# Comparative and Phylogenetic Analyses of the Complete Chloroplast Genomes of Six Almond Species (*Prunus* spp. L.)

**DOI:** 10.1038/s41598-020-67264-3

**Published:** 2020-06-23

**Authors:** Wei Wang, Tao Yang, Hui-Ling Wang, Zhen-Jian Li, Jian-Wei Ni, Shang Su, Xin-Qiao Xu

**Affiliations:** 1 Key Laboratory of Silviculture of the State Forestry Administration, The Institute of Forestry, The Chinese Academy of Forestry, Yi He Yuan Hou, Beijing, 100091 China; 2 Shaanxi Academy of Forestry, Shaanxi, 719000 China; 3 Institute of Forestry and Pomology, Beijing Academy of Agriculture and Forestry Sciences, Beijing, 100097 China

**Keywords:** Evolution, Genetics, Plant sciences

## Abstract

As a source of genetic variation, almond germplasm resources are of great significance in breeding. To better reveal the mutation characteristics and evolution patterns of the almond chloroplast (cp) genome, the complete cp genomes from six almond species were analyzed. The lengths of the chloroplast genome of the six almond species ranged from 157,783 bp to 158,073 bp. For repeat sequence analysis, 53 pairs of repeats (30 bp or longer) were identified. A total of 117 SSR loci were observed, including 96 polymorphic SSR loci. Nine highly variable regions with a nucleotide variability (Pi) higher than 0.08, including *rps16*, *rps16*-*psbK*, *atpF*-*atpH*, *rpoB*, *ycf3*-*rps4*, *rps4*-*ndhJ*, *accD*-*psaI* and *rps7*-*orf42* (two highly variable regions) were located. Based on the chloroplast genome evolution analysis, three species (*P. tenella*, *P. pedunculata* and *P. triloba*) and wild cherry (*P. tomentosa*) were grouped into clade I. Clade II consisted of two species (*P. mongolica* and *P. tangutica*) and wild peach (*P. davidiana*). Clade III included the common almond (*P. dulcis*), cultivated peach (*P. persica*) and GanSu peach (*P. kansuensis*). This result expands the researchers’ vision of almond plant diversity and promotes an understanding of the evolutionary relationship among almond species. In brief, this study provides abundant resources for the study of the almond chloroplast genome, and has an important reference value for study of the evolution and species identification of almond.

## Introduction

The common almond (*Prunus dulcis* (Mill.) D.A.Webb), a member of the family *Rosaceae* and order Rosales, is widely and mainly distributed in the temperate zones of Europe, America, and Asia^1,2^. Among the different varieties of tree nuts, almonds are the second most consumed worldwide, behind peanuts. In 2018, the volume of almonds consumed worldwide amounted to 1.19 million metric tons^1^. In addition to common almonds, there are more than thirty almond species in the world, including partially cultivated and wild varieties^2^. Five wild almond resources have been reported in China, including the diploid species wild almond (*Prunus tenella* Batsch), Mongolic almond (*Prunus mongolica* Maxim.), and Tangut almond (*Prunus tangutica* (Batalin) Koehne), the octoploid species, flowering almond (*Prunus triloba* Lindl.), and the dodecaploid species, longstalk almond (*Prunus pedunculata* (Pall.) Maxim.)^3–6^. All the almond species are highly adapted to a cold and dry climate, which may indicate an important gene pool. Therefore, it is of great significance to identify the almond genetic resources and evaluate their industrial application value for effective utilization and preservation.

Wild almond species usually grow in areas of altitudes between1,100 m and 2,700 m and at latitudes between 28 and 38 N and longitudes between 41 and 54 E^7,8^. It is reported that there are five wild species in China, indicating that China is one of the central origins of almonds^4,5,7^. Based on taxonomy, wild almond species and the common almond were classified as a separate genus, *Amygdalus* L.^9^, outside of the genus *Prunus* L. Subsequently, Gorttapeh also classified the almond resources in the subgenus *Amygdalus* L.^2,10^ as a subgenus of *Prunus*. At present, most authors have suggested putting almond species in the *Prunus* genus rather than considering them as an independent genus^8,11^. Polyploidy exists in the six species, and it is not easy to distinguish between them by evolutionary analysis based on genome sequence and phenotype, which leads to the unclear evolutionary relationships of the wild almond resources^3–6^. However, there is no polyploidy in the chloroplast (cp) genome. In addition, compared with nuclear and mitochondrial genomes, the cp sequence conservation makes it a more common and effective tool for plant phylogenomic analysis.

The chloroplast organelle is the location for photosynthesis and carbon fixation in plants. Because of its unique maternal inheritance and low silent nucleotide substitution rate, chloroplast DNA (cpDNA) has become a useful tool for the study of plant genetic evolution and for interspecific and intraspecific polymorphism identification^12–15^. In addition, the chloroplast is not polyploid, which can be utilized for the genetic analysis of polyploid plants. With the introduction of new sequencing technology, a large amount of DNA sequence data can be obtained at a relatively low cost. The acquisition of DNA sequence data is beneficial for exploring plant evolution and formulating breeding strategies at the molecular level. Because the chloroplast genome evolves slowly relative to the nuclear genome, chloroplast sequences provide valuable resources for studying population genetics, phylogeography, phylogeny, and species identification.

The chloroplast genome is a relatively highly conserved circular DNA with a size ranging from 115 to 165 kb^12–15^. Generally, the cpDNA genome has two large reverse repeat (IR) copies separated by large single-copy (LSC) and small single-copy (SSC) regions. Chloroplast genomes usually contain 110–130 different genes, which have highly conserved gene sequences. Most of them (∼79) encode proteins mainly involved in photosynthesis, while other genes encode approximately thirty transfer RNAs (tRNA) and 4 ribosomal RNAs (rRNA) ^6,12^. The cp genome comparative analysis not only provides information on the genome structure but also plays a significant role in understanding the cp genome evolution, phylogeny and species identification^13–15^.

Here, we present the complete and annotated DNA sequences of the cp genomes of five almond resources. Our research purposes were as follows: (1) to study the overall structure of the cp genomes; (2) to detect the variations in the repeat sequences and the simple sequence repeats (SSRs) in the six almond cp genomes; (3) to screen rapidly evolving DNA regions in the six chloroplast genomes; and (4) to analyze the phylogenetic relationship using the sequence data of the chloroplast genome. These results will supply rich molecular tools for further phylogenetic analysis, population genetics analysis, and species identification and will contribute to almond breeding.

## Materials and methods

### Chloroplast DNA sequencing and genome assembly

Fresh leaves of the almond species were collected from a single plant of each species in different distribution areas (Table 1). Approximately 5 g fresh leaves were harvested as outlined by the improved extraction method^16^ for chloroplast DNA separation. The chloroplast DNA was extracted using the high-salt saline plus Percoll gradient method. After DNA separation, 1 μg of purified DNA was segmented and used to construct a short insertion library (insert size 430 bp) according to the manufacturer’s instructions (Illumina, San Diego, CA, USA)^17^, followed by sequencing on the Illumina HiSeq. 4000.

**Table 1:** Description of the six almond species.

Species	Origin	Collection site	Latitude	Longitude
*P. dulcis* var. ZHIPI	Cultivar	Kashi, Xinjiang, China	38.22	77.18
*P. tangutica*	Wild	Aba, Sichuan, China	32.3	103.38
*P. pedunculata*	Wild	Yulin, Shanxi, China	38.51	109.52
*P. mongolica*	Wild	Alashan, Neimenggu, China	39.46	105.3
*P. tenella*	Wild	Aertai, Xinjiang, China	48.18	86.34
*P. triloba*	Wild	Chicheng, Hebei, China	41.11	116.04

Before assembly, the raw reads were filtered. This filtering step was performed to delete reads with adaptors, reads with a display quality score of less than 20 (Q < 20), reads that contain a percentage of an uncalled base (“N”character) equal to or greater than 10%, and repeat sequences. The reconstruction of the chloroplast genome was based on a combination of *de novo* and reference-guided assemblies. The following three steps were used to assemble the cp genome^18^. First, SOAPdenovo2.04 was used to assemble the filtered reads into contigs^19^. Next, BLAST was used to compare contigs with the reference genome of the six almond species and to align contigs (the similarity and query coverage were more than 80%) according to the comparison genomes. Finally, clean reads were packaged into the assembled draft cp genome to correct the wrong bases, and most of the gaps were filled by the local assembly.

### Genome annotation

The chloroplast genes were annotated with an online DOGMA tool^20^, using the default parameters to predict ribosomal RNA (rRNA) genes, transfer RNA (tRNA) genes, and protein-coding genes. A whole chloroplast genome BLAST search (minimal alignment length percentage > = 40%, E-value < = 1×10^−5^) was performed against five databases^21^: GO^22^ (Gene Ontology), Swiss-Prot^23^, NR (Non-Redundant Protein Database), COG (Clusters of Orthologous Groups)^24,25^, and KEGG (Kyoto Encyclopedia of Genes and Genomes)^26–28^. The chloroplast genome maps of the six almond species were assembled by using OrganellarGenomeDRAW v1.2^29^.

### Repeat sequences and SSRs characteristics

Using REPuter^30^, the size and location of direct (forward), reverse (palindrome), complement and reverse repeat sequences in the chloroplast genomes of the six almond species were identified. For all the repeat types, the hamming distance, equal to the greater sequence identity, in REPuter was equal to 3, or the constraint set was 90%. Using MISA Perl script^31^, simple sequence repeats (SSRs) were detected with thresholds of 10 repeat units for mononucleotide SSRs, six repeat units for dinucleotide SSRs, and five repeat units for tri-, tetra-, penta-, and hexa-nucleotide SSRs.

### Identification of high variable regions

In DnaSP v5.10, based on the alignment sequence matrix of the cp genomes, the sliding window analysis method was used to evaluate the nucleotide variability (Pi) and polymorphic sites (S) with a 200 bp step length and a 600 bp window length^32^. Highly variable regions were defined as the number of polymorphic loci greater than the sum of the average and double the standard deviation. The annotated cp genome determined the locations of these highly variable regions. DnaSP v5.10 was used to evaluate the variable sites, parsimony-informative sites and nucleotide diversity of the hypervariable regions.

### Phylogenomic analysis

To reveal the evolutionary relationship among the six almond species of the *Rosaceae* class, a total of 33 complete cp genomes of the family *Rosaceae* were collected from GenBank, with *Vitis Vinifera* and *Syzygium cumini* as the outer group (Table S1). Using PAUP v4 and 1000 random addition sequences used for a Heuristic search, the phylogenetic trees that were based on maximum parsimony analysis were constructed^33^. The phylogenetic trees based on maximum likelihood analysis were constructed by RAxML-win32-100315, and the bootstrap repetition rate was 1000^34^. The phylogenetic trees based on Bayesian inference was established by MrBayes v3.2.4^35^. The cp genome sequences were compared and visualized by MAFFT v 7.149^36^.

## Results and Discussion

### Genome sequencing and assembly

**Figure 1:**
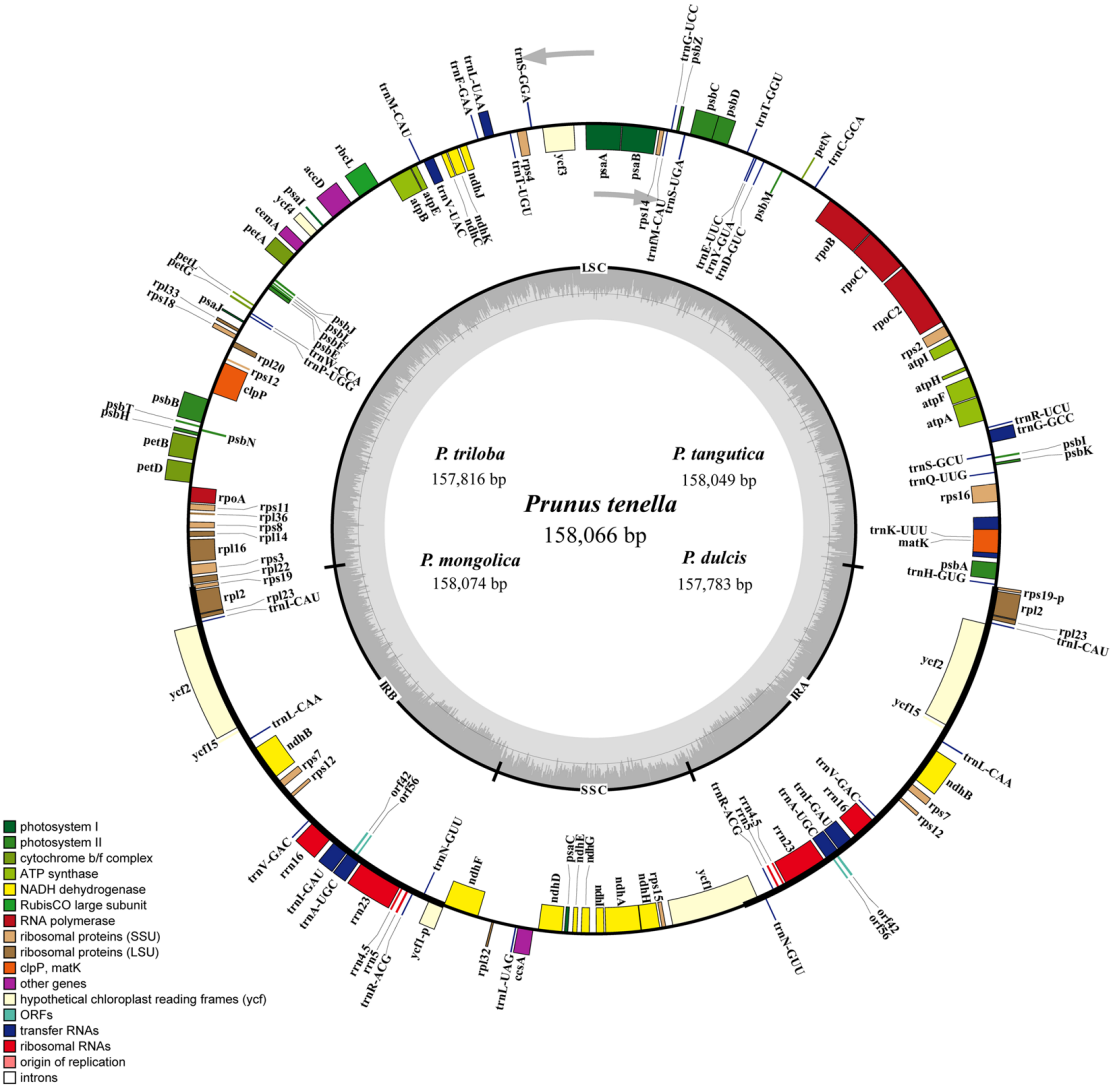
Gene map of the five almond chloroplast genomes. Genes drawn inside the circle are transcribed clockwise, while genes outside the circle are transcribed counterclockwise. The thick lines indicate the extent of the inverted repeats (IRa and IRb) that separate the genomes into large single-copy (LSC) and small single-copy (SSC) regions. The gene functional groups are color-coded.

Among the six almonds species sequenced in this research, 2874 Mb to 4311 Mb of raw data was generated on the Illumina sequencing system, with an average read length of 150 bp. From 2679 Mb to 3995 Mb of reads, the complete chloroplast genome sequence with 16.95× to 25.31× coverage was extracted. Five new almond cp genome sequences were submitted to GenBank (Table 2). The five almond cp genome lengths ranged from 157,783 bp (*P. dulcis* var. ZHIPI) to 158,065 bp (*P*.*tenella*; Fig. 1, Table 2).

**Table 2:** Comparison of the complete chloroplast genome contents of the six almond species.

	P. dulcis	P. pedunculata	P. triloba	P. mongolica	P. tangutica	P. tenella
GeneBank Number	MH727486	MG869261	MH748555	MH727485	MH744156	MH727487
Total Sequence Length	157,783	157,873	157,816	158,074	158,049	158,066
Large Single Copy (LSC)	85,921	86,074	86,021	86,316	86,226	86,260
Small Single Copy (SSC)	19,100	19,029	19,023	18,992	19,063	19,056
Inverted Repeat Region (IR)	26,380	26,384	26,385	26,382	26,379	26,374
Total Number of Gene	136	136	136	136	136	136
Protein-Coding Genes	91	91	91	91	91	91
tRNA	37	37	37	37	37	37
rRNA	8	8	8	8	8	8
GC%	36.77	36.78	36.79	36.76	36.76	36.73
Reference	This article	Wang *et al*. (2019)	This article	This article	This article	This article

### Comparative analysis of the six almond chloroplast genomes

The six almond cp genomes composed of circular double-stranded DNA with a quadripartite structure, consist of an LSC region of 85,921 bp-86,316 bp, an SSC region of 18,992 bp-19,100 bp, and an IR region of 26,374 bp-26,386 bp. The overall GC content of the six almond species was 36.73%-36.79% (Table 2), with a low GC content and a high AT content. Many angiosperm cp genomes have been indicated to be characterized by a low GC content and a high AT content^12,13,37^.

A total of 136 coding genes (Fig. 1, Table 2) were identified, including 91 protein-coding genes, 37 tRNA genes, and eight rRNA genes. Among them, 19 duplicate genes, seven protein-coding genes, eight tRNA genes, and four rRNA genes were found in the IR region. The coding genes of the six almond cp genomes were annotated in the same order.

Among the 136 genes, 21 have one intron (13 protein-coding genes and 8 tRNA genes), and two genes (*clpP* and *ycf3*) have two introns (both protein-coding) (Table 3 & S2, Figure S1). Of the 21 genes with introns, 12 are located in the LSC (eight protein-coding and four tRNAs), four in the IR region (two protein-coding and two tRNAs) and one in the SSC (*ndhA*). One gene, the *rp12* gene, was inversely linked with the duplicated 3’ end exon located in the IR region and the 5’ end exon located in the LSC region. These results indicated that the cp genome structure, the gene number, and the gene order of the six almond species were highly conserved and had similar roles in other genera, such as *Fritillaria*^32^, *Gynostemma*^12^, *Rehmannia*^13^ and *Aconitum*^38^.

**Table 3:** List of genes in the chloroplast genomes of the six almond species.

Category	Gene group	Gene name
*Photosynthesis related genes*	Rubisco	*rbcL*
	Photosystem I	*psaA, psaB, psaC, psaI, psaJ*
	Assembly/stability of photosystem I	*ycf3*(×2)
	Photosystem II	*psbA psbB, psbC, psbD, psbE, psbF, psbH psbI,psbJ, psbK, psbL psbM, psbN, psbT, psbZ*
	ATP synthase	*atpA, atpB, atpE*, ^*a*^*atpF, atpH, atpI*
	Cytochrome b/f complex	*petA*, ^*a*^*petB*,^*a*^*petD, petG, petL, petN*
	Cytochrome c synthesis	*ccsA*
	NADPH dehydrogenase	^*a*^*ndhA*,^*a*^*ndhB*(×2)*, ndhC, ndhD, ndhE, ndhF,ndhG, ndhH, ndhI, ndhJ, ndhK*
Transcription and translation related genes	Transcription	*rpoA, rpoB*,^*a*^*rpoC1, rpoC2*
	Ribosomal proteins	*rps2, rps3, rps4, rps7*(×2)*, rps8, rps11*, ^*c*^*rps12*(×2)*, rps14, rps15*, ^*a*^*rps16, rps18, rps19*, ^*a*^*rpl2*(×2)*, rpl14*, ^*a*^*rpl16, rpl20, rpl22, rpl23*(×2)*, rpl32,rpl33, rpl36*
RNA genes	Ribosomal RNA	*rrn5*(×2)*, rrn4.5*(×2)*, rrn16*(×2)*,rrn23*(×2)
	Transfer RNA	^a^*trnA-UGC*(×2)*, trnC-GCA, trnD-GUC, trnE-UUC, trnF-GAA, trnfM-CAU*, ^a^*trnG-GCC, trnG-UCC, trnH-GUG, trnI-CAU*(×2), ^a^*trnI-GAU*(×2), ^c^*trnK-UUU, trnL-CAA*(×2), ^a^*trnL-UAA, trnL-UAG, trnM-CAU, trnN-GUU*(×2)*, trnP-UGG, trnQ-UUG, trnR-ACG*(×2)*, trnR-UCU, trnS-GCU, trnS-GGA, trnS-UGA, trnT-GGU, trnT-UGU, trnV-GAC*(×2), ^a^*trnV-UAC, trnW-CCA, trnY-GUA*
Other genes	RNA processing	*matK*
	Carbon metabolism	*cemA*
	Fatty acid synthesis	*accD*
	Proteolysis	^*b*^*clpP*
Genes of unknown function	Conserved reading frames	*ycf1, ycf2*(×2)*, ycf4,ycf15*(×2)
	Hypothetical chloroplast protein	*orf42*(×2)*, orf56*(×2)
Pseudogenes		^Ψ^*ycf1*, ^Ψ^*rps19*

^a^ gene containing a single intron, ^b^ gene containing two introns, ^c^ gene divided into two independent transcription units, (×2), gene with two copies; a pseudogene is represented by ^Ψ^.

### Comparison of border regions and sequence identity

**Figure 2:**
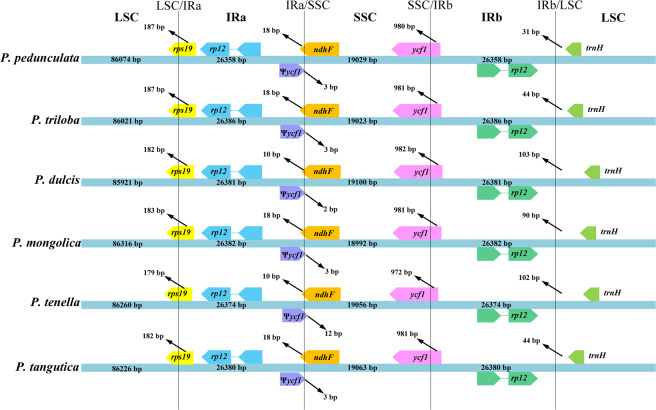
Comparisons of the borders of the large single-copy (LSC), small single-copy (SSC), and inverted repeat (IR) regions among the *P. pedunculata*, *P. triloba*, *P. mongolica*, *P. tenella*, *P. tangutica*, and *P. dulcis* chloroplast genomes. The colored boxes above and below the main line indicate the adjacent border genes, where yellow indicates *rps19*, blue indicates *rpl2*, purple indicates Ѱ*ycf1* (Ѱ indicates a pseudogene), brown indicates *ndhF*, pink indicates *ycf1*, green indicates *rpl2*, and light green indicates *trnH*.

The IR regions of the six cp genomes ranged from 26,358 bp (*P. pedunculata*) to 26,386 bp (*P. triloba*), among which *rps19*, *ycf1*, *ndhF*, *rpl2*, and *trnH* were located at the junction of the LSC/IR and SSC/IR borders. In the expansion and contraction of the IR regions, considerable changes were observed (Fig. 2). For the LSC/IR borders, the IRa/SSC boundary in the almond cp genomes contained some obvious differences (Fig. 2). The gene *rps19* in the LSC of the almond species extended into the IRa region, with a length from 179 to 187 bp. A truncated *ycf1* pseudogene of 984 bp or 1161 bp in size extended 2 bp-12 bp into the SSC region, and overlapped with *ndhF* by 12 bp-22 bp. The gene *trnH* in the LSC region contracted 31–103 bp from the binding region of the IRb/LSC. The length of these six regions and the whole cp genome sequence were affected by the changes in the marginal regions of the IR and the SSC.

**Figure 3:**
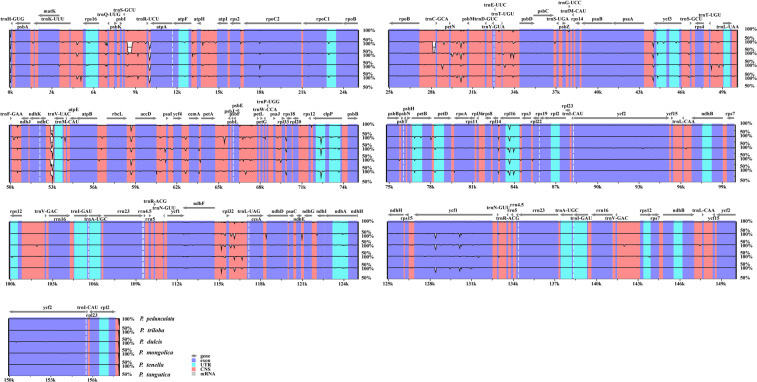
Visualized alignments of the six almond species cp genomes. Sequence identity plots among the five almond species chloroplast genomes were made by using mVISTA, with *P. pedunculata* as a reference. The y-axis represents identity ranging from 50% to 100%.

To indicate the degree of the genome divergence, the sequence identities among the almonds cpDNAs were drawn by mVISTA, with *P. pedunculata* as a reference. The whole sequences showed a high degree of similarity, with only a few areas of less than 90% similarity, indicating that the almonds’ plastomes were quite conserved (Fig. 3). However, a significant divergence was mainly found in the CNS region. As expected, the coding regions had less divergence than the noncoding regions, and the IRs regions were more conservative than the single-copy regions. Similar results have been found in the chloroplast genomes of other genera, such as *Gynostemma*^12^, *Rehmannia*^13^, *Fritillaria*^32^ and *Aconitum*^38^.

### Repeat sequence analysis

**Figure 4:**
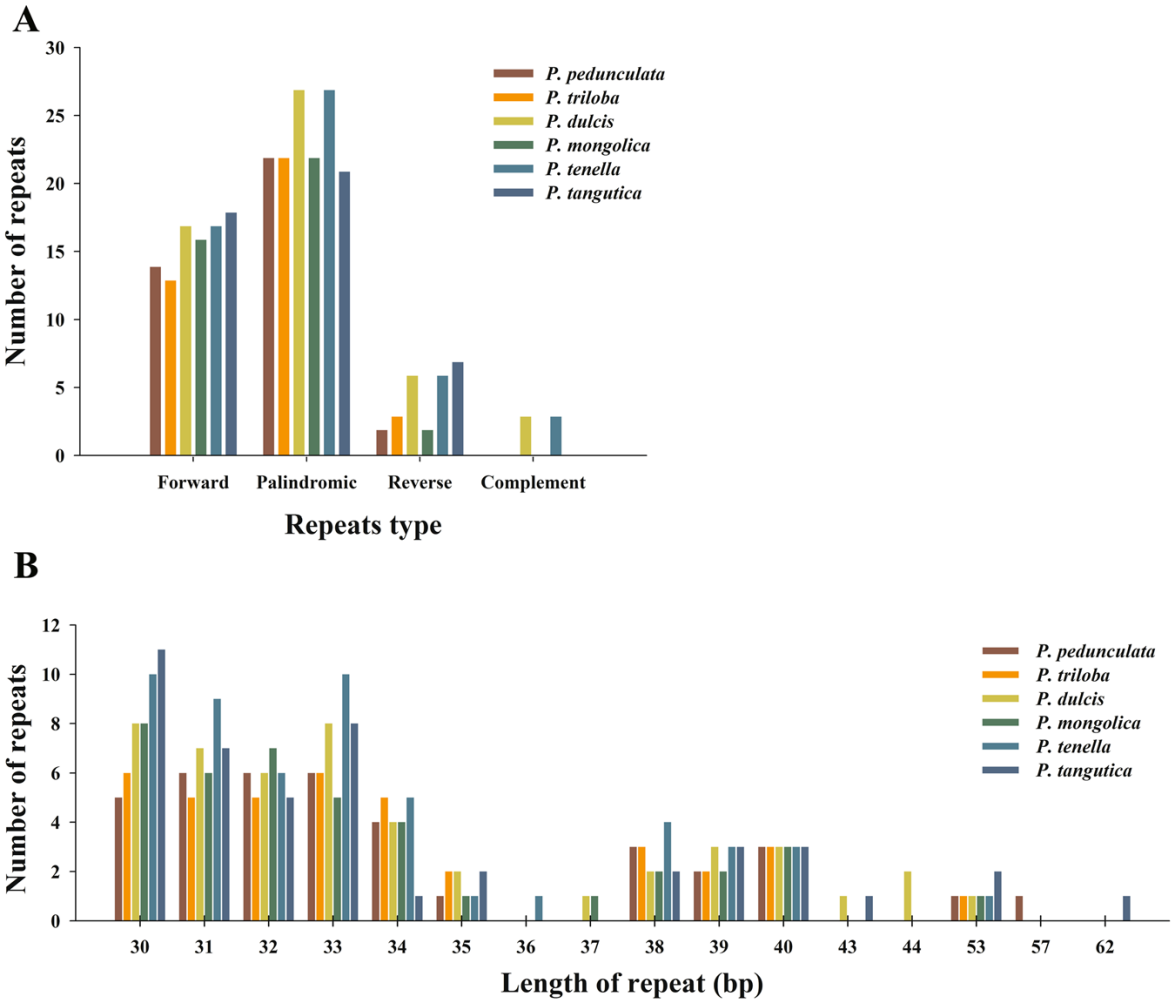
Analyses of repeated sequences in the six almond species chloroplast genomes. (**A**) Frequency of repeat types. (**B**) Frequency of repeat sequences by length.

Repeat motifs play an important part in phylogenetic studies and have important application value in genome rearrangement analysis. In the chloroplast genome of *P. dulcis*, 53 pairs of repeats (30 bp or longer), including 23 palindromic repeats, 17 forward repeats, six reverse repeats, and three complement repeats, were obtained using REPuter (Fig. 4A). In these repeats, one repeat is 53 bp long, two repeats are 44 bp long, one repeat is 43 bp long and 44 are 30–40 bp long (Fig. 4B). Most of these repeats (69.8%) are located in noncoding regions, whereas some are found in genes such as *psaB*, *ndhB*, *ycf1*, *ycf2* and *ycf3* (Table S3). For more information on the six almond species repeat motifs, see Supplementary Table S3.

### Repeat analysis and simple sequence repeats (SSR)

Repeat sequences show high repeatability, high variability, and codominant inheritance in heterozygotes, and are thus effective molecular genetic markers in plant species identification and in evolution and ecology studies^12–16^. An SSR is a repeating unit consisting of 1–6 nucleotides, also known as a short tandem repeat (STR) or a microsatellite. In this research, a large number of SSR loci (Tables S4 & S5) were found by comparing and analyzing the genome sequences of the almond chloroplasts. Five SSR types (mononucleotide, dinucleotide, trinucleotide, tetranucleotide and pentanucleotide repeats) were obtained by comparing the six almond cp genomes; there were no hexanucleotide repeats (Table 4).

**Table 4:** Simple sequence repeats (SSRs) in the six almond cp genomes.

Species	SSR loci no.	PolyM. loci no.	PolyM. loci (100%)	mono-	di-	tri-	tetra-	penta-	Location			Region		
									IGS	Intron	CDS	LSC	IR	SSC
*P. pedunculata*	80	60	75.00	56	14	1	8	1	51	16	13	71	2	7
*P. triloba*	81	60	74.07	58	13	1	8	1	53	16	12	71	2	8
*P. dulcis*	76	55	72.37	52	17	0	5	2	49	15	12	69	3	4
*P. mongolica*	80	57	71.25	53	19	0	6	2	53	16	11	70	2	8
*P. tenella*	85	63	74.12	63	13	0	7	2	56	16	13	73	4	8
*P. tangutica*	75	52	69.33	51	15	0	6	3	50	12	13	65	2	8
Total Loci	117	96	82.05	84	19	2	8	4	80	25	12	100	5	12

A total of 477 SSRs were detected in the almond cp genomes altogether, with each almond cp genome having 76–85 SSRs. These SSRs ranged in length from 10 to 18 bp (Table 4 &S5). There were 117 SSR loci in the chloroplast genomes of the six almond resources, including 96 polymorphic SSR loci. The most abundant SSR type were mononucleotide repeats (333 repeats in 84 loci), followed by dinucleotide repeats (91 repeats in 19 loci), tetranucleotide repeats (40 repeats in 8 loci), pentanucleotide repeats (11 repeats in 4 loci), and trinucleotide repeats (2 repeats in 1 loci) (Table S5). Abundant mononucleotide repeats were also found in *Gynostemma*^12^, *Rehmannia*^13^, *Fritillaria*^32^ and *Aconitum*^38^. Therefore, mononucleotide repeats may have more influence on genetic variation than the other types of SSRs. In this research, mononucleotides almost always consisted of A/T, which was similar to previous results in *Fritillaria*, *Rehmannia* and *Aconitum*. These 117 SSR loci were located less frequently in the IR regions (5 SSRs) and mainly in the LSC region (100 SSRs), followed by the SSC region (12 SSRs; Table S5). Only one SSR locus extended over the IRA/SSC boundary, which was located in the protein-coding gene *ycf1* in the cp genome of *P. dulcis* var. ZHIPI. In addition, the SSRs in the almond cp genomes were mainly located in the intergenic spacers (IGS; 80 SSRs), followed by the introns (25 SSRs), with a minority in the coding DNA sequences (CDS; 12 SSRs). The SSR loci in the CDS regions of the almond cp genome were distributed in nine protein-coding genes (*matK*, *rpoC2*, *rpoB*, *atpB*, *cemA*, *rps18*, *ndhE*, *ndhI*, and *ycf1*) (Table S5). Yu *et al*.^32^ also observed that 20 different SSRs of the *Fritillaria* cp genome were distributed in nine protein-coding genes (*matK*, *rpoC1*, *rpoC2*, *cemA*, *ndhD*, *ndhG*, *ndhH*, *ycf2*, and *ycf1*), but the nine protein-coding genes were not identical. Lu *et al*.^39^ found that 15 different SSRs were located in eight protein-coding genes (*ycf1*, *cemA*, *rpoC2*, *ycf2*, *ndhH*, *rpl22*, *ndhD*, and *ndhE*) of three *Cardiocrinum* chloroplast genomes. The SSR loci can be used for phylogenic study and species identification when the SSRs in the plant chloroplast genomes show abundant variation.

### Recognition of highly variable regions

The highly variable region of the chloroplast genome can provide important information for phylogeny research, which can be used to identify closely related species more accurately^32,40^.

**Figure 5:**
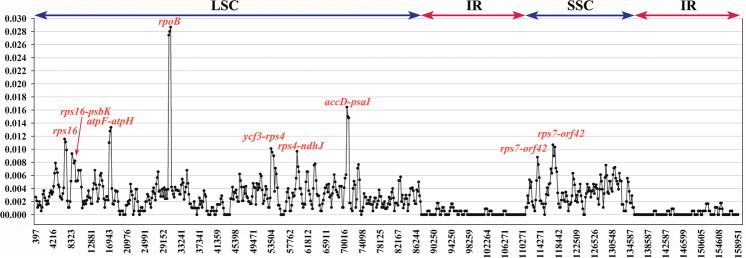
Sliding window analyses of the six almond species chloroplast genomes sequences. The X-axis represents the position of the midpoint of a window, while the Y-axis represents nucleotide variability (Pi) of each window (window length: 600 bp, step size: 200 bp).

As shown in Fig. 5, the sliding window in the DnaSP 5.0 software accurately located nine highly variable regions with nucleotide variability (Pi) greater than 0.008, including *rps16*, *rps16*-*psbK*, *atpF*-*atpH*, *rpoB*, *ycf3*-*rps4*, *rps4*-*ndhJ*, *accD*-*psaI* and *rps7*-*orf42* (two highly variable regions). The highest Pi value, for *rpoB*, is 0.02867. Two of nine highly variable regions were located in the SSC region, and seven of these regions were located in the LSC region; the nucleotide diversity values in the IR regions were not higher than 0.002, and no highly differentiated sequence was detected, indicating that these regions were highly conserved. The sequences of these highly variable regions will provide a valuable resource for research in phyletic evolution, population genetics, species identification, and breeding directions.

### Phylogenetic analysis based on the cp genomes

**Figure 6:**
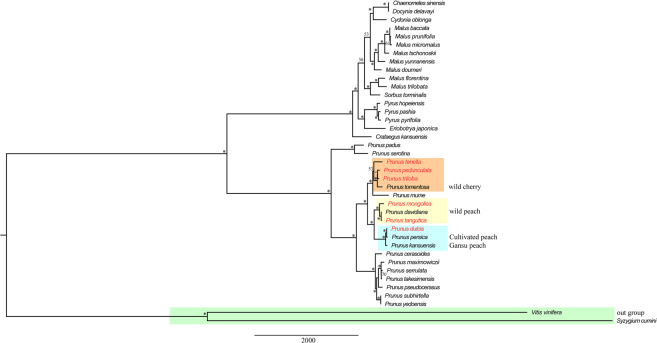
Phylogenetic relationships of 39 species inferred from maximum likelihood (ML) analysis based on the complete chloroplast genome sequences. The numbers above the nodes are the support values with maximum likelihood (ML) analyses, and the symbol * indicates that the support rate of the branch is 100%. Clade I (orange): *P. tenella*, *P. pedunculata, P. triloba* and *P. tomentosa*; clade II (yellow): *P. mongolica*, *P. tangutica* and *P. davidiana*; clade III (light blue): *P. dulcis*, *P. persica* and *P. kansuensis*.

The phylogenetic trees were constructed from four data partitions of the cp genome of 39 *Rosaceae* plants: the complete cp genome (Fig. 6, Figure S3 A, and Figure S4 A), the LSC (Figure S2 A, Figure S3 B, and Figure S4 B), the SSC (Figure S2 B, Figure S3 C, and Figure S4 C), and the IR (Figure S2 C, Figure S3 D, and Figure S4 D). The complete cp genome was highly conserved, and the best resolution of the phylogenetic relationship can only be obtained by using the complete cp genome sequences. Therefore, we mainly discuss the phylogenetic relationships according to Fig. 6. All of the *Rosaceae* were clustered into three main high support groups (Fig. 6). The six almond species in this study were clustered into one large group, in which the six *Amygdalus* resources were divided into three small clades. Three species (*P. tenella*, *P. pedunculata* and *P. triloba*) and wild cherry (*P. tomentosa*) were grouped into clade I. Clade II included two species (*P. mongolica* and *P. tangutica*) and wild peach (*P. davidiana*). Clade III consisted of *P. dulcis*, *P. persica* (cultivated peach) and *P. kansuensis* (Gansu peach). The results of the chloroplast genome cluster analysis showed that the resources of the subgenus *Amygdalus* were clearly classified. Similar results were obtained by using RAxML-win32-100315 software (Figures S3 A) and MrBayes v3.2.4 software (Figures S4 A) for the same evolutionary analysis of the complete cp genomes. If the evolutionary analysis was not based on the complete chloroplast genome sequences, then no consistent evolutionary relationship could be obtained (Figure S2; Figure S3 B-D; Figure S4 B-D).

The relationships of the *Rosaceae* plants have been well resolved in previous studies^41,42^, but the status of almonds remains uncertain. The resources in clade I are clustered together, which is consistent with their high latitude geographical distribution. The results of clade II are consistent with the genome-based cluster analysis, in which common almond (*P. dulcis*) is closely related to the cultivated peach (*P. persica*), and the Mongolic almond and Tangut almond is the closest to the cultivated peach in wild resources^41,43^. These results indicate that the common almond (*P. dulcis*) and Tangut almond (*P. tangutica*) belong to the same genus as the peach. The genome of *P. pedunculata* and *P. triloba* are polyploid, which is difficult to distinguish based on the evolutionary analysis of genome sequence and phenotype, which leads to an unclear evolutionary relationship of the wild almond resources. In this study, the cluster analysis showed that wild almond (*P. tenella*), longstalk almond (*P. pedunculata*) and flowering almond (*P. triloba*) are closely related and cluster together with wild cherry.

Whether almond resources, including the six almond resources in this study, belong to the independent genus *Amygdalus* L. or the genus *Prunus* L. has always been a complicated matter^2,9,10^. Based on the phenotypic classification of classical botany, it is very difficult to clearly divide the genetic relationship of the six species. From the perspective of chloroplast clustering in this study, wild almond (*P. tenella*), longstalk almond (*P. pedunculata*) and flowering almond (*P. triloba*) should be classified into the genus *Prunus* L., while the common almond (*P. dulcis*), Mongolic almond (*P. mongolica*) and Tangut almond (*P. tangutica*) should be divided into the subgenus *Amygdalus* L.

## Conclusions

In this study, we report the chloroplast genomes of five almond species by *de novo* sequencing and compare them with one previously published almond cp genome sequence. These six complete almond cp genomes showed the most common genomic characteristics but still provided abundant genetic information for the sequence differentiation and structure research of the almond species. For repeat sequence analysis, 53 pairs of repeats (30 bp or longer) were identified. The 117 SSR loci were observed. Nine highly variable regions with nucleotide diversity were located. Large repeat sequences, SSRs, and highly variable regions provided possible sequence information for genetic markers. Genetic markers were used for the identification of plant germplasm resources and the improvement of plant fingerprints. A phylogenetic tree was constructed with the complete cp genomes to better understand the genetic relationships of the almond species. Three species (*P. tenella*, *P. pedunculata* and *P. triloba*) and wild cherry (*P. tomentosa*) were group into clade I. Clade II consisted of two species (*P. mongolica* and *P. tangutica*) and wild peach (*P. davidiana*). Clade III included common almond (*P. dulcis*), cultivated peach (*P. persica*) and GanSu peach (*P. kansuensis*). Phylogenetic trees not only improve the classification of almonds but also provide guidance for selecting parents with nearer genetic relationships and hybridization compatibility in almond breeding research. These results expand the researchers’ vision of almond plant diversity and promote an understanding of the evolutionary relationship among almond species. In brief, this study provides abundant resources for the study of the chloroplast genome of almonds, and has important reference value for studies on the evolution and species identification of almonds.

## Supplementary information

Supplementary Information.

Supplementary Information 2.

Supplementary Information 3.

Supplementary Information 4.

Supplementary Information 5.

Supplementary Information 6.

Supplementary Information 7.

Supplementary Information 8.

Supplementary Information 9.

Supplementary Information 10.
