# Supplementary figures and images for: Comparative and Phylogenetic Analyses of the Complete Chloroplast Genomes of Six Almond Species (Prunus spp. L.)
Source: Sci Rep. 2020 Jun 23;10:10137. doi: 10.1038/s41598-020-67264-3 (PMC7311419; doi:10.1038/s41598-020-67264-3)

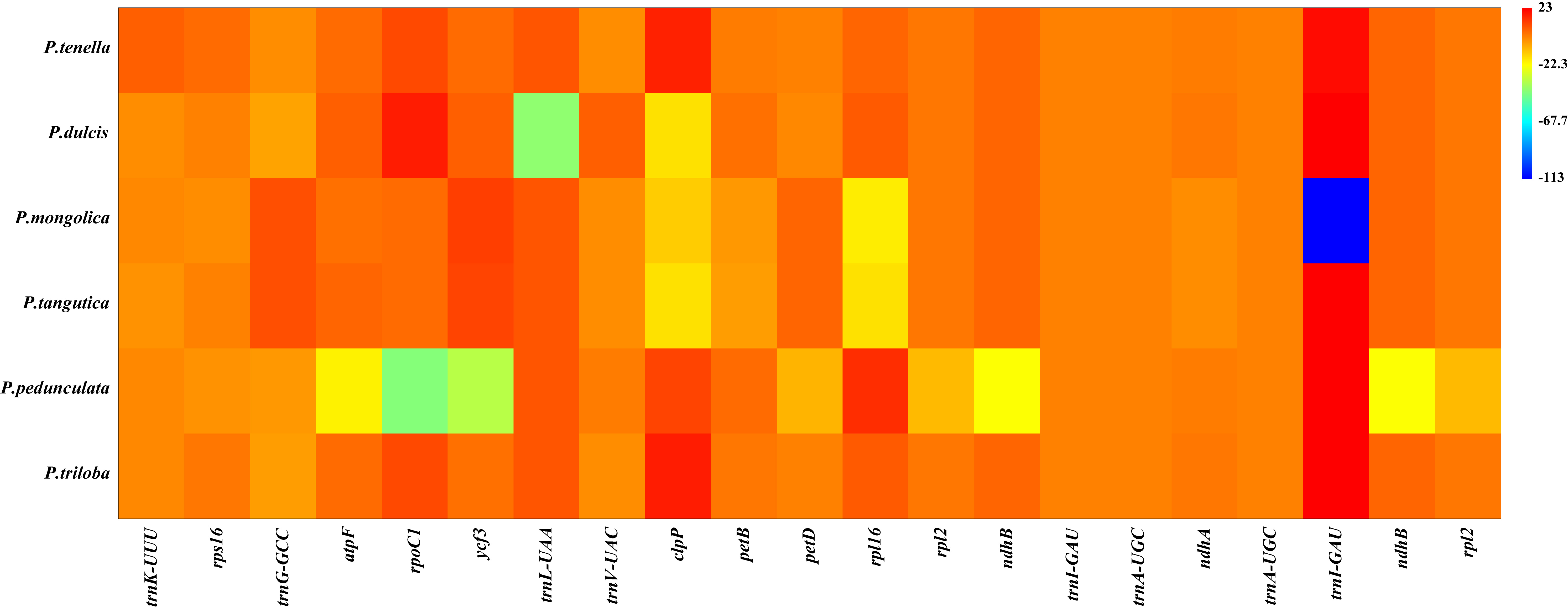

Supplement: Supplementary file 2 — Supplementary Information 2. [file 41598_2020_67264_MOESM2_ESM.jpg]

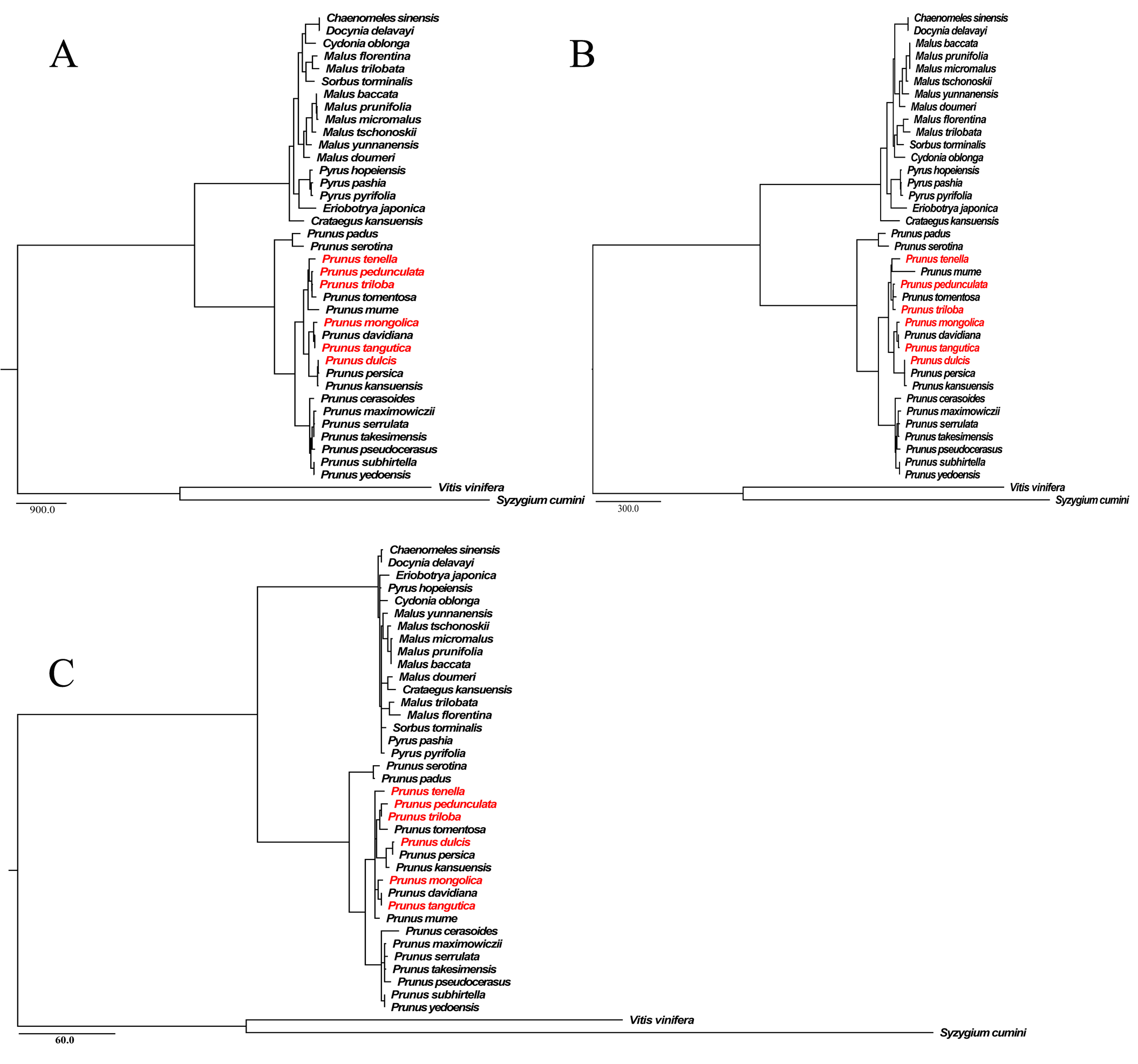

Supplement: Supplementary file 3 — Supplementary Information 3. [file 41598_2020_67264_MOESM3_ESM.tif]

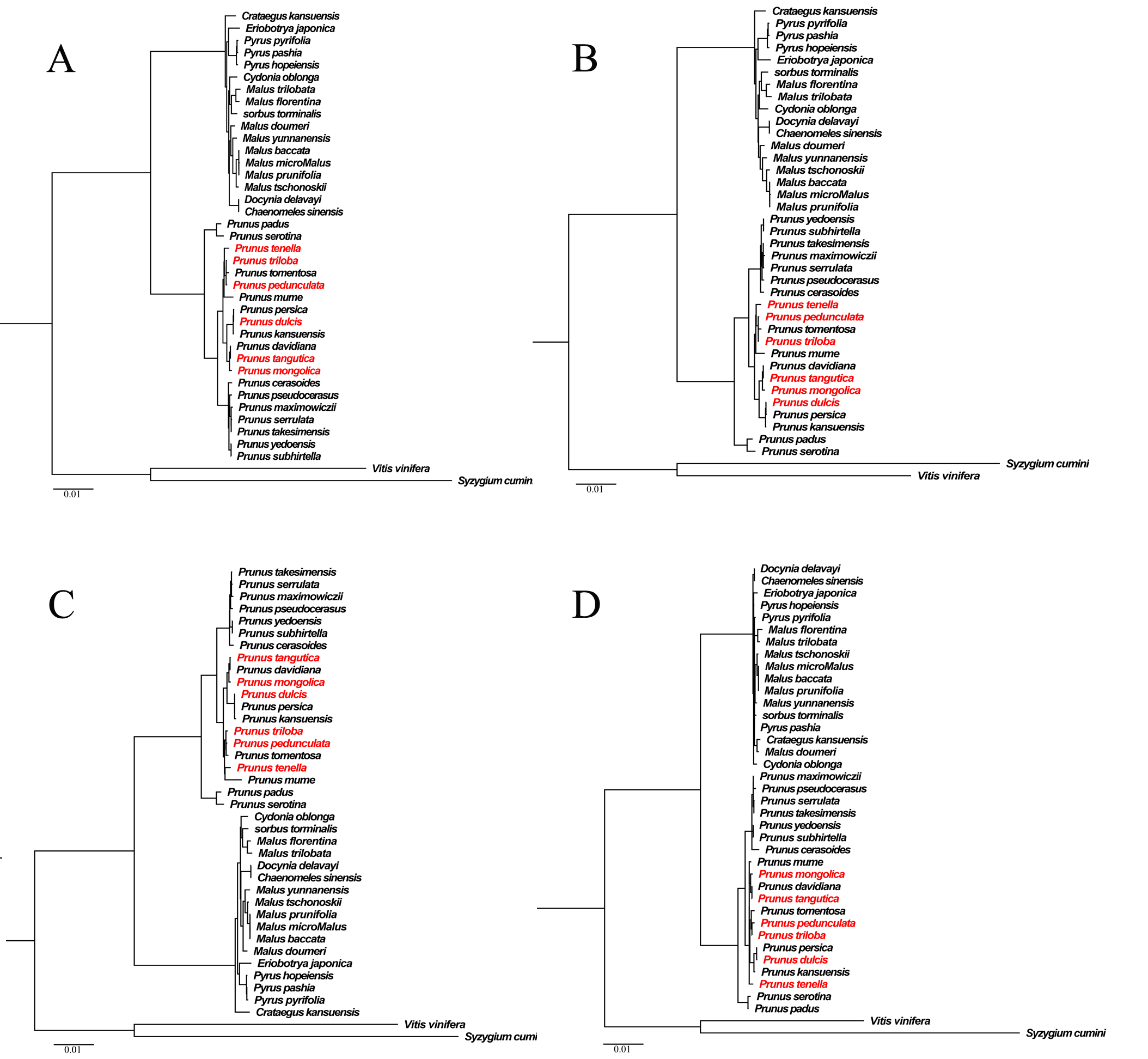

Supplement: Supplementary file 4 — Supplementary Information 4. [file 41598_2020_67264_MOESM4_ESM.tif]

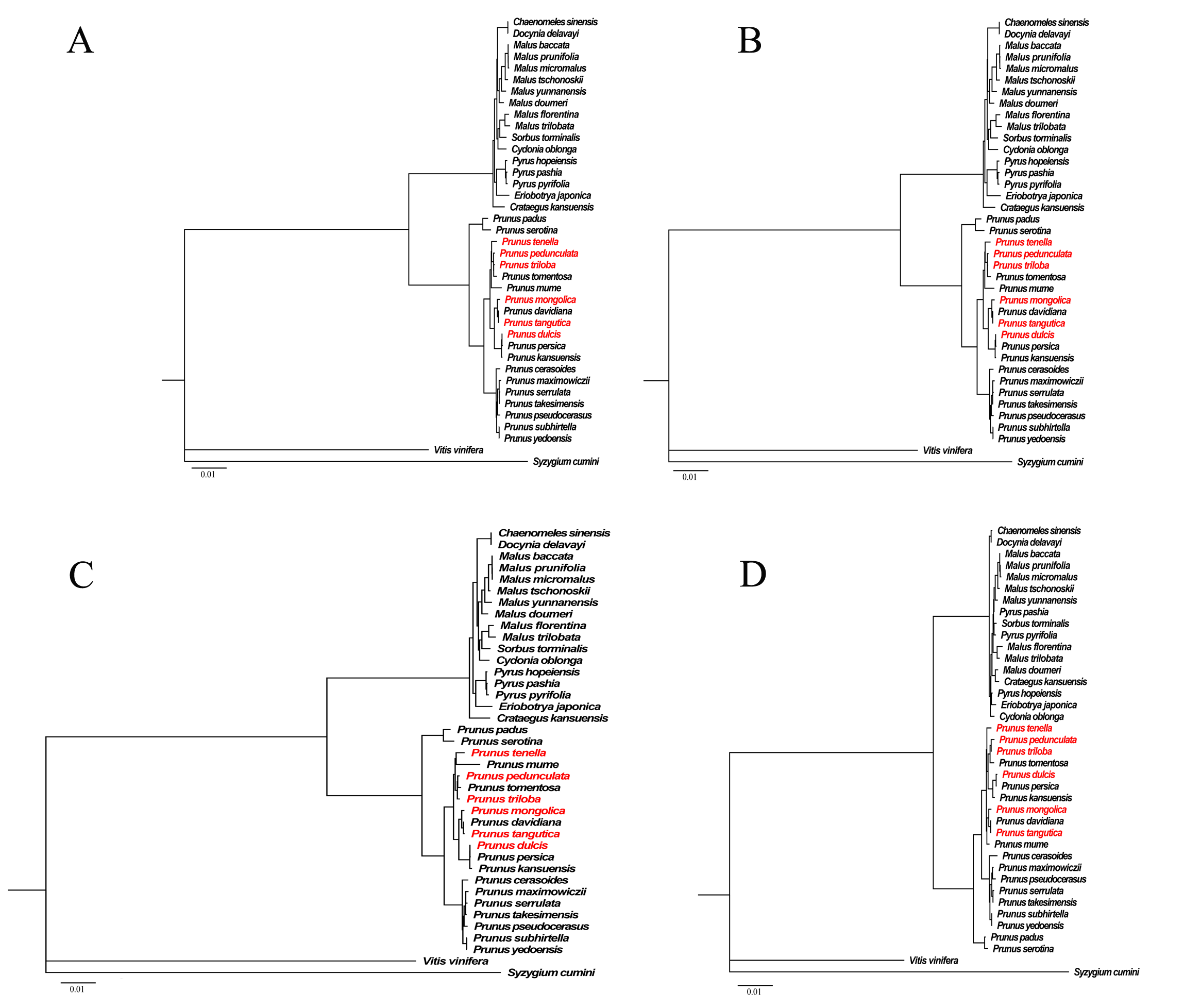

Supplement: Supplementary file 5 — Supplementary Information 5. [file 41598_2020_67264_MOESM5_ESM.tif]
