# Supplementary material for: Comparative and Phylogenetic Analyses of the Complete Chloroplast Genomes of Six Almond Species (Prunus spp. L.)
Source: Sci Rep. 2020 Jun 23;10:10137. doi: 10.1038/s41598-020-67264-3 (PMC7311419; doi:10.1038/s41598-020-67264-3)
Supplement: Supplementary file 6 — Supplementary Information 6. [file 41598_2020_67264_MOESM6_ESM.docx]

Figure S1 Heatmap of length changes in the introns in six almond chloroplast genomes. From red to blue, the cell color reflects the variation of intron length from low to high.

Figure S2 Using PAUP v4 software, the phylogenetic relationships of 39 were species inferred from maximum likelihood (ML) analyses of different data partitions. (A) LSC region. (B) SSC region. (C) IR region.

Figure S3 Using RAxML-win32-100315 software, the phylogenetic relationships of 39 species were inferred from maximum likelihood (ML) analyses of different data partitions. (A) Complete chloroplast genome. (B) LSC region. (C) SSC region. (D) IR region.

Figure S4 Using MrBayes v3.2.4 software, the phylogenetic relationships of 39 species were inferred from Bayesian (BI) analyses of different data partitions. (A) Complete chloroplast genome. (B) LSC region. (C) SSC region. (D) IR region.
